# Inflammasomes as contributors to periodontal disease

**DOI:** 10.1002/JPER.20-0157

**Published:** 2020-08-06

**Authors:** Julie T. Marchesan

**Affiliations:** ^1^ Department of Comprehensive Oral Health Periodontology Adams School of Dentistry University of North Carolina Chapel Hill NC

**Keywords:** AIM2, alveolar bone loss, caspase inhibitors, caspase‐1, IFI16, Ifi204, inflammasome, periodontitis

## Abstract

A genome‐wide association study of ≈2.5 million markers identified unique biologically informed periodontal complex traits with distinct microbial communities and interleukin‐1β (IL‐1β) levels. Each trait was associated with different single nucleotide polymorphisms. These variants include genes associated with immune responses, microbial colonization, and the epithelial barrier function. The specific set of variants leads to individual biological paths that converge into an overlapping clinical phenotype of periodontal tissue destruction. This concept suggests that periodontal disease is a group of distinct conditions. We identified polymorphisms in inflammasome genes *interferon gamma inducible protein 16* (*IFI16*) and *absent in melanoma 2* (*AIM2*) that were associated with increased severity of periodontal disease. Inflammasomes respond to pathogen or tissue “danger” signals and assemble into multiprotein “machineries” that are essential for the cleavage of proinflammatory mediator IL‐1β into an active form. Thus, understanding how variants of *IFI16* and *AIM2* contribute to periodontal disease pathogenesis may lead to treatment options that address individual biological variations and precision therapies for oral health.

## 1 VARIABILITY IN SUSCEPTIBILITY TO PERIODONTAL DISEASE

Periodontitis is known for being a complex, multifactorial inflammatory disease. Evidence suggests that periodontal disease is actually a group of different biological conditions with overlapping clinical presentations.^1^ Each of these conditions is influenced by several genetic variants, ultimately resulting in a destructive inflammatory response against the omnipresent microbial biofilm.

**FIGURE 1:**
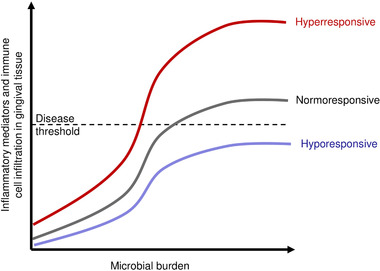
Variation in the subgingival inflammatory response upon the same microbial challenge. Schematic representation of differences in inflammatory mediator production and immune cell infiltration among individuals affects the response to gingival microbial burden. The middle line represents individuals with a normal response. The top line represents hyperresponsive individuals who would exhibit clinical symptoms of disease at a lower threshold of microbial burden. The bottom line represents hyporesponsive individuals who would be relatively resistant to periodontitis. Inspired by Champagne et al. 2003^2^

It is already well documented that some individuals have a lower than average inflammatory response to bacterial challenge in the periodontium, and others have a higher than average inflammatory response (Fig. 1).^2^ Studies published since the 1980s show that not everyone is equally susceptible to periodontitis. A 15‐year study of Sri Lankan tea laborers with abundant plaque and calculus and without access to dental care showed three distinct groups: ≈11% had no progression beyond gingivitis, ≈81% had moderate progression, and ≈9% had rapid progression of periodontal disease.^3^ Thus, severity of disease is not a simple function of plaque accumulation. Despite this understanding of individual variations in the periodontal inflammatory response and disease progression, treatments targeting host deficiencies are still limited for treating periodontitis. Such treatments could fundamentally change how periodontitis is treated, in particular by preventing the progression of disease and potentially decreasing the need for non‐surgical and surgical treatments.

Additional studies showed that, even among patients who were treated for periodontal disease and are under maintenance care, a subset (16% to 22%) of individuals would progress to moderate‐to‐severe disease^4^, ^5^ (and reviewed by Kornman and collaborators^6^). Understanding the distinct biology of individuals that develop periodontal disease and those that are resistant would help clinicians determine different types of adjunct treatments. This approach is in line with the concept of precision medicine in which prevention and treatment strategies take individual biological variability into account.^7^ Indeed, new classification systems for periodontal disease, such as the 2018 update to the American Academy of Periodontology (AAP) periodontal disease classification,^8^ that provide more granular and precise diagnostic parameters may facilitate determination of causes of individual variation.

## 2 USING GWAS TO EXPLORE PERIODONTAL DISEASE SUSCEPTIBILITY

**FIGURE 2:**
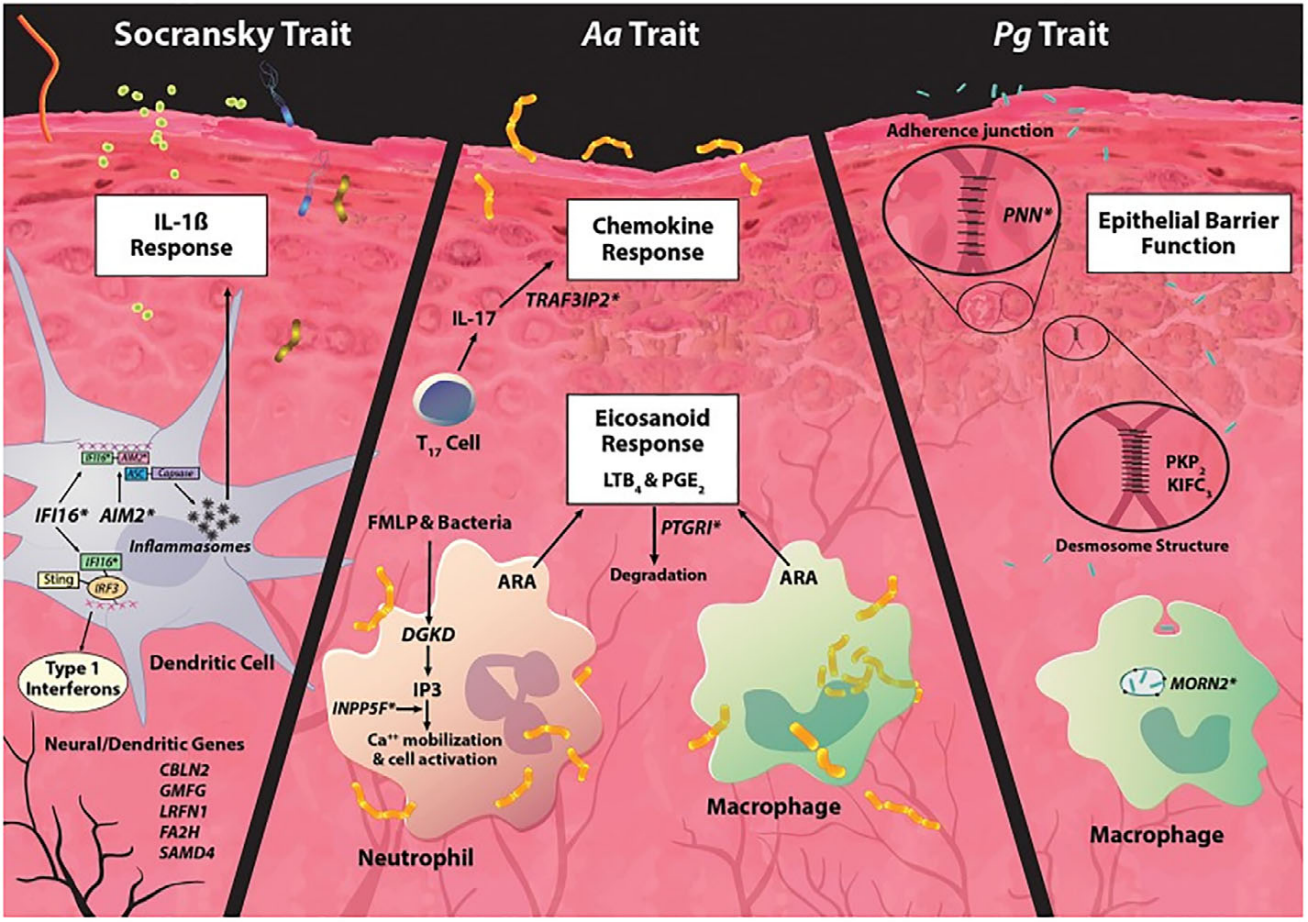
Characteristics of the three main periodontal complex traits used to identify loci associated with periodontal disease susceptibility. Reprinted with permission from Offenbacher et al. (2016).^1^ *SNPs significantly associated with a periodontal complex trait (PCT).

A strategy for understanding biological differences among individuals is genome‐wide association study (GWAS) analysis. These types of studies can identify single nucleotide polymorphisms (SNPs) that are associated with disease development and severity and have been used as an approach to help understand multiple diseases. Initial efforts using only a clinical criteria of chronic periodontitis have had only modest success in identifying any SNPs that were significantly associated with disease.^9^ However, a unique approach defined disease as periodontal complex traits (PCTs) by supplementing clinical data with biological intermediates of microbial burden (eight periodontal pathogens) and the local inflammatory response by interleukin 1β (IL‐1β) in the gingival crevicular fluid (GCF).^1^ This approach led to identification of genetic loci significantly associated with several unique biologically informed PCTs. Three main PCTs were identified^1^ (Fig. 2): PCT1, called the Socransky trait, is characterized by the presence of a high number of periodontal pathogens and a moderate amount of GCF‐IL‐1β; PCT3, named the *Aggregatibacter actinomycetemcomitans* (*Aa)* trait, is characterized by high amounts of IL‐1β and dominated by the periodontal pathogen *Aa*; and PCT5, named the *Porphyromonas gingivalis* (*Pg)* trait, is dominated by the periodontal pathogen *Pg*.

In addition to the unique biological characterization, the loci significantly associated with each PCT were also distinct. The Socransky trait was associated with SNPs that interfered with genes encoding *interferon gamma inducible protein 16* (*IFI16*) and *absent in melanoma 2* (*AIM2*), proteins that form inflammasomes, and lead to increased levels of mature IL‐1β upon a microbial challenge. The *Aa* trait correlated with polymorphisms in genes that affect the processing of prostaglandin and IL‐17. The *Pg* trait was associated with SNPs in genes that influence the epithelial barrier function, including the gene encoding plakophilin 2 (PKP2), which is present within desmosomes of epithelial cells. We suggest that any functional impairment in the epithelial boundary adjacent to the subgingival biofilm has the potential to increase the risk for disease. Other traits include PCT6, which is most strongly associated with clinically determined disease and has the highest plaque levels and the highest association with smoking and Type 2 diabetes mellitus.^1^ Complex traits PCT7‐PCT11 are associated with health and lower clinical plaque scores and have microbial community structures dominated by low counts of Gram‐negative periodontal microorganisms *Prevotella nigrescens, Treponema denticola, Tannerella forsythia*, and *Prevotella intermedia*. Intriguingly, most of the loci associated with each of these biological traits had not previously been explored in the pathogenesis of periodontal disease.^1^

## 3 INFLAMMASOMES IN PERIODONTAL DISEASE

**FIGURE 3:**
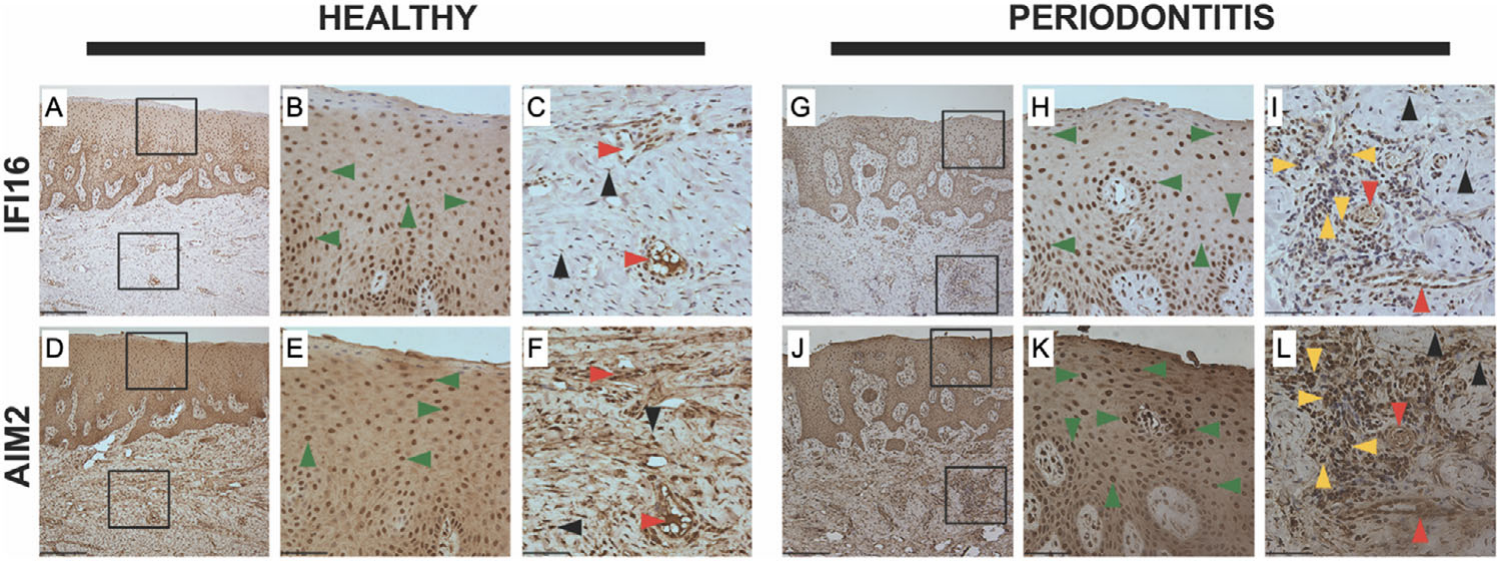
Immunohistochemical detection of IFI16 and AIM2 in human gingival tissues. Images of tissue sections from a healthy individual (**A through F**) and an individual with periodontal disease (**G through L**) according to the American Academy of Periodontology classification,^26^ stained with antibodies recognizing the indicated proteins. A, D, G, J represent original magnification × 10 (scale bar = 200 mm); B, C, E, F, E, H, K, I, L represent original magnification × 40 (scale bar = 50 mm) of the square inserts located in the figures with original magnification × 10 in the epithelial and connective tissue layer. Green arrowheads = epithelial cells; black arrowheads = fibroblasts; yellow arrowheads = leukocytes; red arrowheads = endothelial cells

Six genes had multiple polymorphisms that were significantly associated with the Socransky trait.^1^ Among these genes, *IFI16* and *AIM2* are part of an SNP‐containing region on chromosome 1 that encodes multifunctional proteins involved in innate immunity. Until the GWAS finding, IFI16 had never been explored in the context of periodontal disease. Analysis of the locus revealed the presence of two haplotype blocks.^10^ Compared with individuals without the SNPs in either of these haplotype blocks, individuals within the blocks had high levels of periodontal microorganisms (50‐ to 200‐fold higher numbers of specific pathogens), higher IL‐1β in the GCF, and increased clinical parameters of periodontal disease. Analysis of gingival tissues from healthy individuals and subjects with periodontal disease confirmed that both IFI16 and AIM2 were present in multiple cells of the periodontium, including epithelial cells, fibroblasts, and endothelial cells (Fig. 3).^10^ Additionally, migrating neutrophils expressing IFI16 and AIM2 were observed in the epithelial layer in gingival tissues derived from individuals with periodontal disease, further supporting a potential role of these proteins in the pathogenesis of periodontitis.^11^

**FIGURE 4:**
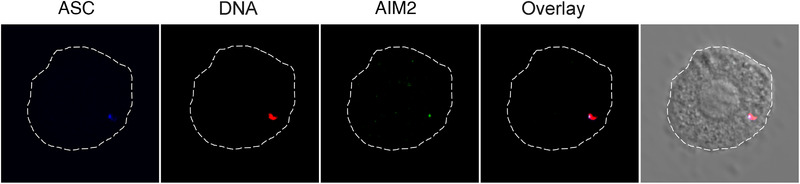
Visualization of inflammasome activation by recognition of cytosolic DNA. Murine dendritic cells were primed with lipopolysaccharide then stimulated with rhodamine‐labeled poly‐dAdT DNA resulting in Aim2 inflammasome activation. Confocal images show an overlay of pseudocolored ASC, a protein recruited to inflammasomes (blue), DNA (red), and AIM2 (green) in the cytosol of a cell. Reprinted with permission from Marchesan et al. (2020)^12^

**FIGURE 5:**
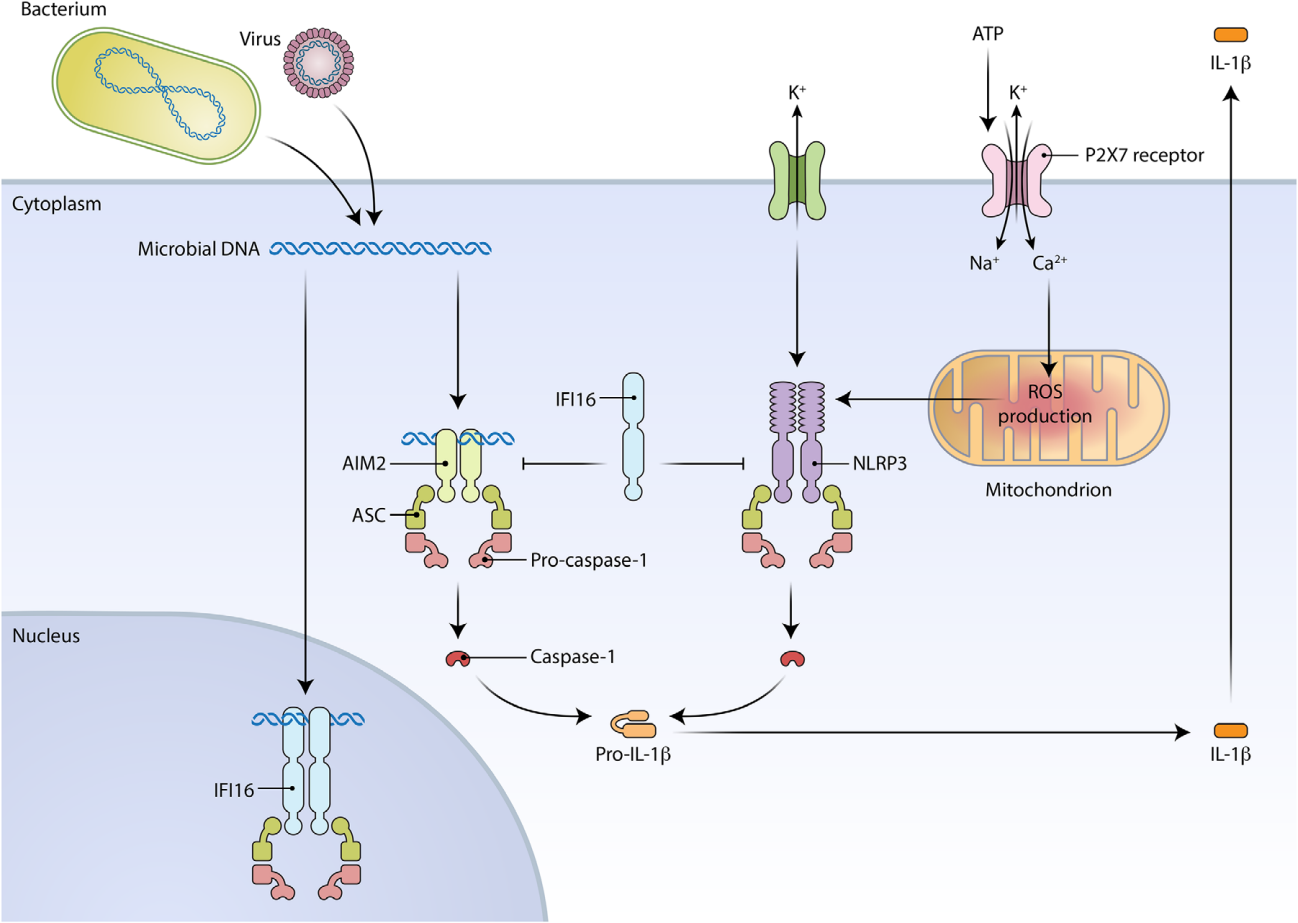
Proinflammatory and anti‐inflammatory functions of AIM2 and IFI16. IFI16 and AIM2 form inflammasome complexes that respond to microbial DNA to promote production of mature IL‐1β. IFI16 also inhibits AIM2 and NLRP3 inflammasome activity to limit production of mature IL‐1β. The priming step that induces expression of the inflammasome genes and *IL1B* is not shown. [Credit: Heather McDonald, BioSerendipity, LLC, Elkridge, MD]

Both IFI16 and AIM2 are members of the PYHIN family of proteins, which all have PYrin and HIN domains. Both have DNA‐binding domains and are pattern‐recognition receptors that function as the sensor component of protein complexes called inflammasomes.^12^, ^13^, ^14^ Inflammasomes are an essential component of the two‐step process by which functional proinflammatory IL‐1β is produced. In response to activation of cell surface receptors, genes encoding the IL‐1β precursor and components forming the inflammasome are induced (named cell priming or signal 1). After priming, a second‐specific signal will activate individual inflammasome (like IFI16 and AIM2) and lead to the formation of unique inflammasome complexes with caspase‐1 that cleave the IL‐1β precursor into an active form. AIM2 functions as an inflammasome sensor in the cytoplasm (Fig. 4),^14^, ^15^ whereas IFI16 functions as an inflammasome sensor in the cytoplasm and nucleus.^16^, ^17^ In these roles, AIM2 and IFI16 are proinflammatory (Fig. 5). However, depending on the pathogenic encounter, IFI16 can also have inflammasome‐antagonizing functions by suppressing AIM2 and NLRP3 activities (Fig. 5).^18^, ^19^

**FIGURE 6:**
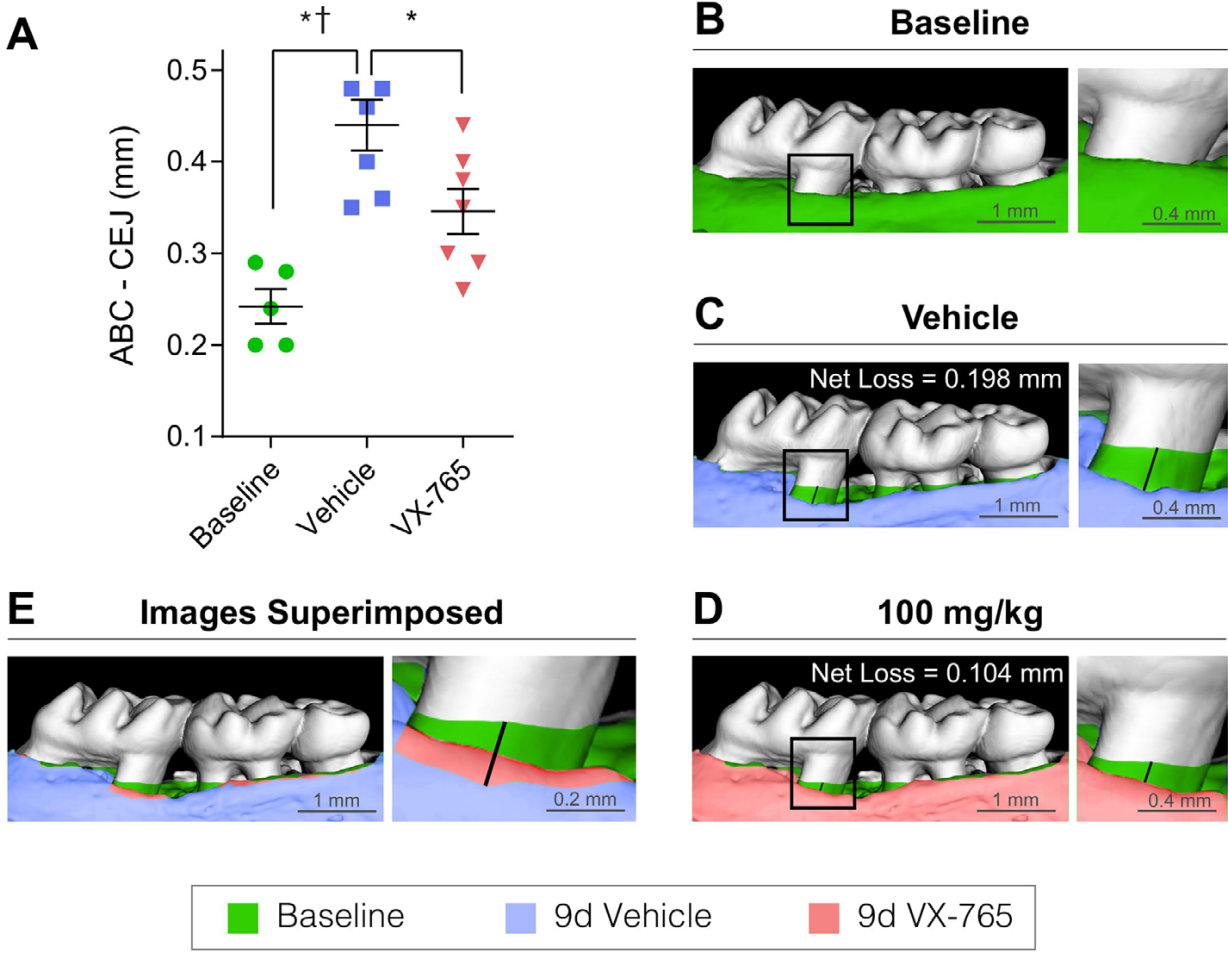
Caspase‐1 inhibition blocks ≈50% of alveolar bone loss in mice at 9 days of experimental periodontitis. Starting 1 day before induction of experimental periodontitis with the ligature model,^20^ mice received a twice daily oral dosage of the caspase‐1 inhibitor VX‐765 (100 mg/kg) for 10 days. Ligatures were placed between M1 and M2 and kept until the end of the experiment (9 days total). **A)** Measurements taken from the alveolar bone crest to the cementum‐enamel junction show significant inhibition of alveolar bone loss compared with vehicle control. **B**) Representative image shows murine maxilla at baseline on the day before ligature placement (alveolar bone represented in green). **C**) Representative image shows maxilla from vehicle‐treated mouse at day 9 after ligature placement (bone represented in blue, baseline in green superimposed). **D**) Representative image shows maxilla from VX‐765‐treated mouse at day 9 after ligature placement (bone represented in pink). **E**) Superimposed maxilla at baseline (green), 9‐day VX‐765‐treated mice (pink), and 9‐day vehicle (blue) shows the amount of bone loss during the 9‐day experimental periodontitis. Adapted from Marchesan et al. (2020).^12^ *<0.05, ^†^<0.01

Further analysis by our group showed that multiple genes encoding inflammasome components, including *Aim2*, *Ifi204* (the mouse homolog of *IFI16*), and *Nlrp3*, exhibit increased expression in gingival tissues of murine experimental periodontitis. In addition, gingival tissues show increased expression of proinflammatory *Il1b*.^12^, ^20^ To block inflammasome formation, our group administered the oral caspase‐1 inhibitor VX‐765 to mice with ligature‐induced periodontitis. The results showed that inhibition of caspase‐1 led to an ≈50% reduction in alveolar bone loss, further supporting a major role of inflammasome formation in inflammatory‐driven bone loss (Fig. 6).^12^

Targeting of IL‐1β has been explored as a therapy for several diseases, which creates an opportunity for repurposing such drugs to treat other inflammatory diseases.^12^ Blocking IL‐1β production with VX‐765 as an orally administered drug has been previously tested in clinical trials for epilepsy^21^ and psoriasis (clinical trial: NCT00205465). Preclinical studies indicate that it may also be useful in Alzheimer disease^22^ and some forms of arthritis.^23^ No increased rates of infection have been reported in studies with this inhibitor, which has been reported as a concern for blocking IL‐1β to treat inflammatory diseases.^24^ Thus, VX‐765 may represent a clinical strategy to limit periodontal disease progression in individuals that have a hyperinflammatory response as the biological basis of disease. Because caspase‐1 is common to multiple types of inflammasomes, identification of receptors that dominate individual responses would be ideal to limit specific inflammatory pathways. Evidence supporting the predisposition of groups of individuals to develop periodontitis is evolving.^1^, ^6^ Additionally, inflammasome polymorphisms were recently shown to put men at greater risk for developing periodontitis.^25^ Further understanding of the role of the inflammasome in disease pathogenesis will help lead to precision care. Based on our knowledge thus far, individuals within the Socransky trait may benefit by adjunct therapeutic inhibition of the inflammasome to treat periodontitis.
